# Intraspecific variation in thermal acclimation and tolerance between populations of the winter ant, *Prenolepis imparis*

**DOI:** 10.1002/ece3.6229

**Published:** 2020-04-08

**Authors:** Maria Adelena Tonione, So Mi Cho, Gary Richmond, Christian Irian, Neil Durie Tsutsui

**Affiliations:** ^1^ Department of Environmental Science, Policy, and Management University of California‐Berkeley Berkeley CA USA; ^2^ Present address: Department of Preventive Medicine Yonsei University College of Medicine Seoul Korea; ^3^ Present address: Department of Family Health Care Nursing UCSF School of Nursing San Francisco CA USA

**Keywords:** adaptation, chill‐coma recovery, climate change, knockdown time, phenotypic plasticity, thermal tolerance

## Abstract

Thermal phenotypic plasticity, otherwise known as acclimation, plays an essential role in how organisms respond to short‐term temperature changes. Plasticity buffers the impact of harmful temperature changes; therefore, understanding variation in plasticity in natural populations is crucial for understanding how species will respond to the changing climate. However, very few studies have examined patterns of phenotypic plasticity among populations, especially among ant populations. Considering that this intraspecies variation can provide insight into adaptive variation in populations, the goal of this study was to quantify the short‐term acclimation ability and thermal tolerance of several populations of the winter ant, *Prenolepis imparis*. We tested for correlations between thermal plasticity and thermal tolerance, elevation, and body size. We characterized the thermal environment both above and below ground for several populations distributed across different elevations within California, USA. In addition, we measured the short‐term acclimation ability and thermal tolerance of those populations. To measure thermal tolerance, we used chill‐coma recovery time (CCRT) and knockdown time as indicators of cold and heat tolerance, respectively. Short‐term phenotypic plasticity was assessed by calculating acclimation capacity using CCRT and knockdown time after exposure to both high and low temperatures. We found that several populations displayed different chill‐coma recovery times and a few displayed different heat knockdown times, and that the acclimation capacities of cold and heat tolerance differed among most populations. The high‐elevation populations displayed increased tolerance to the cold (faster CCRT) and greater plasticity. For high‐temperature tolerance, we found heat tolerance was not associated with altitude; instead, greater tolerance to the heat was correlated with increased plasticity at higher temperatures. These current findings provide insight into thermal adaptation and factors that contribute to phenotypic diversity by revealing physiological variance among populations.

## Graphical abstract

Cold and heat tolerance and acclimation ability differed among populations of the winter ant (*Prenolepis imparis*). High‐elevation populations displayed increased tolerance to the cold and less plasticity. However, heat tolerance was not associated with altitude; instead, greater heat tolerance was correlated with greater plasticity.

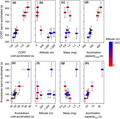

## 1 INTRODUCTION

One of the most substantial drivers of biodiversity loss is climate change (Sala et al., 2000). When novel climatic conditions are physiologically strenuous, species are driven to adapt via genetic change, to migrate, to persist via physiological plasticity (i.e., acclimation), or to succumb to extinction (Fuller et al., 2010). Migration to new suitable habitat is possible, yet difficult for most species (Parmesan & Yohe, 2003). Of the potential outcomes that stressed species will face, adaptation and plasticity are the only options that do not involve local extinction.

Adaptation to new conditions requires organisms to possess genetic architecture that can respond to natural selection in a relatively short time. Although there are examples of rapid heritable genetic changes in populations in response to climate change (Bradshaw & Holzapfel, 2008), most organisms’ life spans are too long and climate change occurs too rapidly. Within this short time frame, an organism's susceptibility to new environmental conditions (i.e., “tolerance”) can be buffered by plasticity of fitness‐related traits (Huey et al., 2012; Seebacher, White, & Franklin, 2015; Somero, 2010). Understanding how species will respond to changing conditions requires understanding the extent of plasticity in fitness‐related traits in natural populations (Bozinovic, Calosi, & Spicer, 2011; Calosi, Bilton, & Spicer, 2008; Fuller et al., 2010; Seebacher et al., 2015). Intraspecific variation in thermal tolerance has been well‐documented in several *Drosophila* species, including *D. buzzatti* and *D. melanogaster* (Sarup, Frydenberg, & Loeschcke, 2009; Sgrò et al., 2010), as well as other organisms (e.g., the common killifish (*Fundulus heteroclitus*; Fangue, Hofmeister, & Schulte, 2006), Collembola (Bahrndorff, Loeschcke, Pertoldi, Beier, & Holmstrup, 2009), and tsetse fly (*Glossina pallidipes*; Terblanche, Clusella‐Trullas, Deere, & Chown, 2008)). Variation in traits among populations within a species can provide insights into adaptive variation in thermal tolerance (Somero, 2002) as well as ecological factors that contribute to evolution of a species in nature (McKechnie, Freckleton, & Jetz, 2006). In this study, we examined the patterns of thermal tolerance (resistance to both heat and cold stress) and plasticity (variation in tolerance after prior exposure to heat or cold) as our fitness traits.

When a species is distributed across a heterogeneous environment, its populations undergo local adaptation. This is often manifested in novel physiological adaptations, tolerance, or acclimation capacities. Such changes commonly occur in species that occupy ecological gradients such as latitudinal and altitudinal clines (Bozinovic et al., 2011). Local adaptation in thermal limits can be detected when populations are raised over successive generations in a common environment (Hoffmann, Sørensen, & Loeschcke, 2003; Somero, 2010). Such approaches can reveal whether patterns in variation in thermal tolerance is the result of developmental acclimation, maternal effects, or genetic variation (Hoffmann & Sgrò, 2017; Kawecki & Ebert, 2004).

Local adaptation can be beneficial for individuals in predictable environments; however, for populations that are in unpredictable or rapidly changing environments, an ability to acclimate might become maladaptive. For example, Kristensen et al. (2008) found that cold‐acclimated fruit flies (*Drosophila melanogaster)* were better able than flies that were susceptible to the cold at locating resources at low temperatures in the field. Yet, such advantage was lost in warm temperatures, indicating acclimation to the cold came at a cost. Phenotypic plasticity can be costly as it requires energy and flexibility on many different biological scales (Auld, Agrawal, & Relyea, 2010; Murren et al., 2015). Several studies have found an inverse relationship between stress resistance and the capacity for plasticity, suggesting an evolutionary trade‐off. For example, Stillman (2003) found Porcelain crabs (genus *Petrolisthes*) with the highest upper thermal limits also had the lowest acclimation ability. In this case, the evolution of basal thermal tolerance has occurred at the expense of plasticity in thermal tolerance. This is often seen in organisms that are already near their thermal limits (Hoffmann, Chown, & Clusella‐Trullas, 2013; Stillman, 2003; Sunday, Bates, & Dulvy, 2011). However, other studies have found no such trade‐off (Calosi et al., 2008; Gunderson & Stillman, 2015; Kellett, Hoffmann, & McKechnie, 2005) and some have even found the opposite pattern; for instance, increased upper thermal limits in diving beetles (genus *Deronectes*) were associated with a greater ability to acclimate (Calosi et al., 2008).

Ants have proven to be extremely useful model systems for monitoring environmental impacts: They are abundant, widespread, ecologically vital, sensitive to environmental stress, and relatively easy to collect (Ribas, Campos, Schmidt, & Solar, 2012). For ants, temperature is the primary constraining force in determining seasonal activities (Dunn, Parker, & Sanders, 2007; Netherer & Schopf, 2010). Tolerance of extreme temperature in ants has been positively linked to body size (Verble‐Pearson, Gifford, & Yanoviak, 2015).

The winter ant (*Prenolepis imparis*; Say, 1836) is particularly well suited for studying responses to temperature changes. It is found across a large elevational gradient in California, from sea level to high elevation, and thus provides an opportunity to study mechanisms of thermal adaptation in a wide range of natural populations. This species is often associated with cooler microhabitats in mesic forests (Cuautle, Vergara, & Badano, 2016; Frye & Frye, 2012; Wheeler, 1930), and it decreases foraging in response to warmer temperatures (Stuble et al., 2013). Worker ants are usually highly abundant and behaviorally dominant when colonies are actively foraging (Fellers, 1987, 1989; Lessard, Dunn, & Sanders, 2009; Lynch, Balinsky, & Vail, 1980)*.* Activity of *P. imparis* is also quite seasonal, often low during the warmer months and high in cooler months (early spring and late fall) when most other ant species exhibit reduced foraging (Dunn et al., 2007). This preference for cooler temperatures and capacity to survive in a broad geographic range make the winter ant an ideal candidate to study plastic responses to thermal stress.

In this study, we examined natural populations of *P. imparis* to illustrate patterns of thermal tolerance (resistance to both heat and cold stress) and plasticity (variation in tolerance after prior exposure to heat or cold). To assess tolerance, we quantified responses to thermal stress by measuring heat knockdown time and chill‐coma recovery time (CCRT) as indicators of heat and cold tolerance, respectively (Maysov & Kipyatkov, 2009). We included these performance tests because they are simple and robust measures of tolerance (Angilletta et al., 2007; Overgaard & MacMillan, 2017). To examine plasticity, we quantified short‐term reversible acclimation (Piersma & Drent, 2003) and analyzed these data in the contexts of altitude and body size. We also measured the thermal characteristics of *P. imparis* habitat across geographically dispersed sites and related these conditions to the observed physiological responses to thermal stress.

## 2 MATERIALS AND METHODS

**FIGURE 1:**
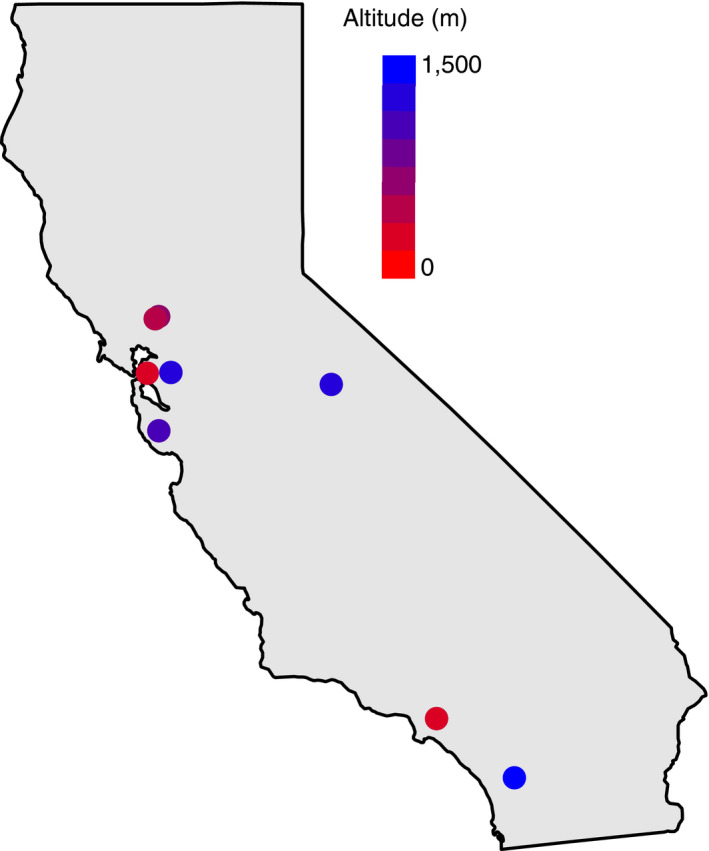
Map of California with sampled localities. Localities colored according to elevation

We chose populations of *P. imparis* for performance tests from sites across different elevations within California, USA (Figure 1). Ants were collected via aspirator, primarily during January, February, and March 2015. Samples from one population (Mt. Diablo) were collected in March 2017. Elevation and GPS coordinates were taken at all locations using a Garmin GPS (WGS1984; Table 1). All statistical analyses were performed in R version 3.4.0 (R Core Team, 2017) in RStudio 1.0.143 (RStudio Team, 2015).

**Table 1:** For each locality, altitude (m), GPS coordinates, mean mass, chill‐coma recovery times (CCRT ± *SE*), and knockdown times (±*SE*) after a 10°C and 27°C acclimation are reported

Population	Longitude	Latitude	Alt (m)	Mass^a^ (mg)	Mean CCRT			Mean knockdown		
					10°C^b^	27°C^b^	Acclimation capacity	10°C^b^	27°C^b^	Acclimation capacity
Berkeley	−122.26317	37.87281	71	1.00	174 ± 9	744 ± 36	570	41 ± 5	87 ± 11	46
Whittier	−118.05395	34.00381	100	0.78	180 ± 9	768 ± 38	588	60 ± 7	127 ± 15	67
Stebbins	−122.09678	38.50867	109	0.90	189 ± 9	807 ± 39	618	50 ± 6	106 ± 13	56
Quail Ridge	−122.14895	38.48307	388	0.88	149 ± 7	636 ± 30	487	46 ± 6	98 ± 12	52
Castle Rock	−122.09495	37.22829	973	0.82	137 ± 6	583 ± 28	446	47 ± 6	101 ± 12	54
Mt Diablo	−121.916667	37.219167	1,130	0.83	144 ± 7	616 ± 29	472	48 ± 6	102 ± 13	54
Yosemite	−119.58584	37.74763	1,233	1.28	143 ± 10	612 ± 39	469	85 ± 11	182 ± 22	97
Palomar	−116.92146	33.34078	1,442	0.95	145 ± 7	619 ± 32	474	46 ± 6	99 ± 12	53

Notes

Units for CCRT and knockdown are reported in seconds as calculated from predicted means. Acclimation capacity is also reported across the different populations.

^a^ Indicates mean dry weight of individuals tested.

^b^ Acclimation temperature.

### 2.1 Field temperatures

To assess the thermal environment of the ants’ habitat, we collected microclimate data at a subset of localities. At these sites, we measured ambient and underground temperatures once per hour from January 2015 to August 2017: We placed iButtons (DS1922L, Maxim Integrated) in the field two meters above each ant nest and below ground at the minimum depth that we expected to find nesting chambers (60 cm; Tschinkel, 1987). Additionally, we used this depth to record underground temperatures that were less susceptible to daily fluctuations (Parton & Logan, 1981). We calculated the mean temperature and standard deviation at each site for each month using the individual hourly temperature readings obtained for that month over multiple years, when applicable. We then used these monthly means to calculate the annual means.

### 2.2 Performance tests

Only nonreplete foragers (workers that do not have enlarged abdomens due to food storage) were collected for use in knockdown trials. To acclimate these individuals to a constant temperature, we divided them into two separate 20‐cm‐diameter plastic tubs containing a dish of 20% sugar water solution and two nesting chambers. Each tub contained approximately 125 ants when the collection numbers allowed (Appendix S1). One plastic tub was placed in an incubator (CAT# 11‐690‐650D, Fisher Scientific) at 27°C (warm‐acclimated), and the other was placed in a growth chamber (CMP3246; Conviron) at 10°C (cold‐acclimated). Both treatments were kept in the dark for the entire acclimation period. To reduce positional effects of acclimation temperature, ant tubs were periodically rearranged within the chamber or incubator. Dead ants were removed, and sugar water was replaced every three days. The ants were kept in these conditions for at least seven consecutive days, after which we performed thermal tolerance assays.

To quantify cold tolerance, we measured CCRT, defined as the time required for ants to resume an upright position after exposure to a temperature low enough to induce a chill coma (Macmillan, Williams, Staples, & Sinclair, 2012). We used this timing as a measure of tolerance because it has been previously demonstrated to correlate with minimum temperature (Andersen et al., 2015). Yet, it is worth noting that this is a measurement of resistance to the effects of cold rather than tolerance. Nevertheless, it is commonly used as a measure of thermal tolerance and especially useful for examining relative differences (Sinclair, Coello Alvarado, & Ferguson, 2015). After acclimating the ants to 10°C or 27°C, as described above, we covered them in ice for three hours which, in preliminary trials, was the amount of time required for all individuals to enter a chill coma, but not so long that they were unable to recover. To do this, we enclosed ants in a glass Petri dish with a glass lid, which was placed on ice in a Styrofoam cooler. We then placed ice on top of the dish and closed the cooler. After the prespecified time, we removed the ants from the ice‐surrounded dish and immediately placed them on their backs in a 140‐mm Petri dish at room temperature (~22°C). We recorded CCRT for each ant. We included five replicates of ten ants for each population when collection numbers allowed (Appendix S1). Ants that did not recover after 25 min at room temperature were considered as having incurred chill injury (Castañeda, Lardies, & Bozinovic, 2005). We recorded these data as 25 min, with a delta of one and flagged as being right‐censored (see Section 2.3).

For heat tolerance assays, we acclimated the ants to 10°C or 27°C. We then placed five replicates of ten cold‐ or warm‐acclimated ants on a 140‐mm Petri dish with Insect‐A‐Slip (BioQuip)‐coated sides. The Petri dish was floated in a prewarmed water bath (Fisher Scientific Isotemp Digital‐Control Water Bath). We continuously monitored the surface of the Petri dish with a self‐adhesive thermocouple (SA1‐T‐SRTC; Omega) and maintained the temperature at 43°C ± 1°C. This temperature was obtained from preliminary trials in which the ants did not knockdown immediately and experienced knockdown within fifteen minutes (data not shown). The time for ants to collapse on the surface of the Petri dish due to a loss of coordination was recorded as the knockdown time for each ant.

After thermal testing, all ants used for CCRT and heat knockdown trials were frozen on dry ice and preserved in 100% ethanol. The dry weight of these samples was taken after evaporating the ethanol in a drying oven for at least one hour. Ethanol preservation will change the mass of the individuals; however, because all individuals were stored the same way, their masses can be compared within this study (Knapp, 2012).

### 2.3 Data analysis

We used accelerated failure time models to analyze both knockdown and chill‐coma trials using the *survreg* function in the “survival” package v2.42‐3 (Therneau, 2016; Therneau & Grambsch, 2000). This model was chosen over the more commonly used Cox proportional hazards (PH) to overcome the violation of proportionality of hazards rates (Williams et al., 2014). For our data, “survival” corresponded to “remaining standing” versus “remaining in chill coma.” In the model, we included “delta” which corresponds to survival after the temperature trial. In order to accentuate the among‐population and environmental effects, we used a mixed effects model. Time until recovery (for chill‐coma trials) or time until knockdown was used as a dependent variable. As fixed explanatory variables, we included acclimation temperature and population of origin, while biological replicate was included as a random effect. Significance of fixed effects was assessed using the *ANOVA* function in the “car” package (Fox et al., 2019). For post hoc tests, we then compared combinations of population of origin and acclimation temperature against the null hypothesis (the differences between the effects is zero) using the *glht* function in “multcomp” package (Hothorn, Bretz, & Westfall, 2008), adjusting for false discovery rates (Tukey). For all analyses, significance was taken at the level *p* < .05. We simplified the model by removing statistically nonsignificant interactions and conditions until no further simplification was possible. Then, we chose the most parsimonious model with the lowest Akaike information criterion (AIC) among exponential, Weibull, Gaussian, logistic, lognormal, and loglogistic error distributions (Lebreton, Burnham, Clobert, & Anderson, 1992). To account for skewed distributions of heat knockdown times and CCRT, we reported the predicted means as calculated by the *predict* function in the “survival” package (Therneau, 2016). We employed the calculated means to quantify the level of thermal plasticity in response to acclimation. For each population, we calculated the cold‐ and heat‐specific acclimation capacity individually similar to Kellett et al. (2005) and van Heerwaarden, Kellermann, and Sgrò (2016):

$$\begin{array}{c} {\text{acclimation capacity}}_{\text{cold}}=\left(\text{mean CCRT at}\;27^{\circ }\text{C acclimation}\right)-\left(\text{mean CCRT at}\;10^{\circ }\text{C acclimation}\right)\\ {\text{acclimation capacity}}_{\text{hot}}=\left(\text{mean knock down time at}\;27^{\circ }\text{C acclimation}\right)-\left(\text{mean knock down time at}\;10^{\circ }\text{C acclimation}\right) \end{array}$$

A smaller *acclimation capacity_cold_* (faster recovery to the cold) and larger *acclimation capacity_hot_* (able to resist the heat) indicate a better ability to phenotypically adjust to a changing environment (i.e., greater plasticity).

To determine the relationship between thermal tolerance and elevation, body size, and plasticity levels, we used linear regression. We applied a Bonferroni correction for multiple tests.

## 3 RESULTS

**FIGURE 2:**
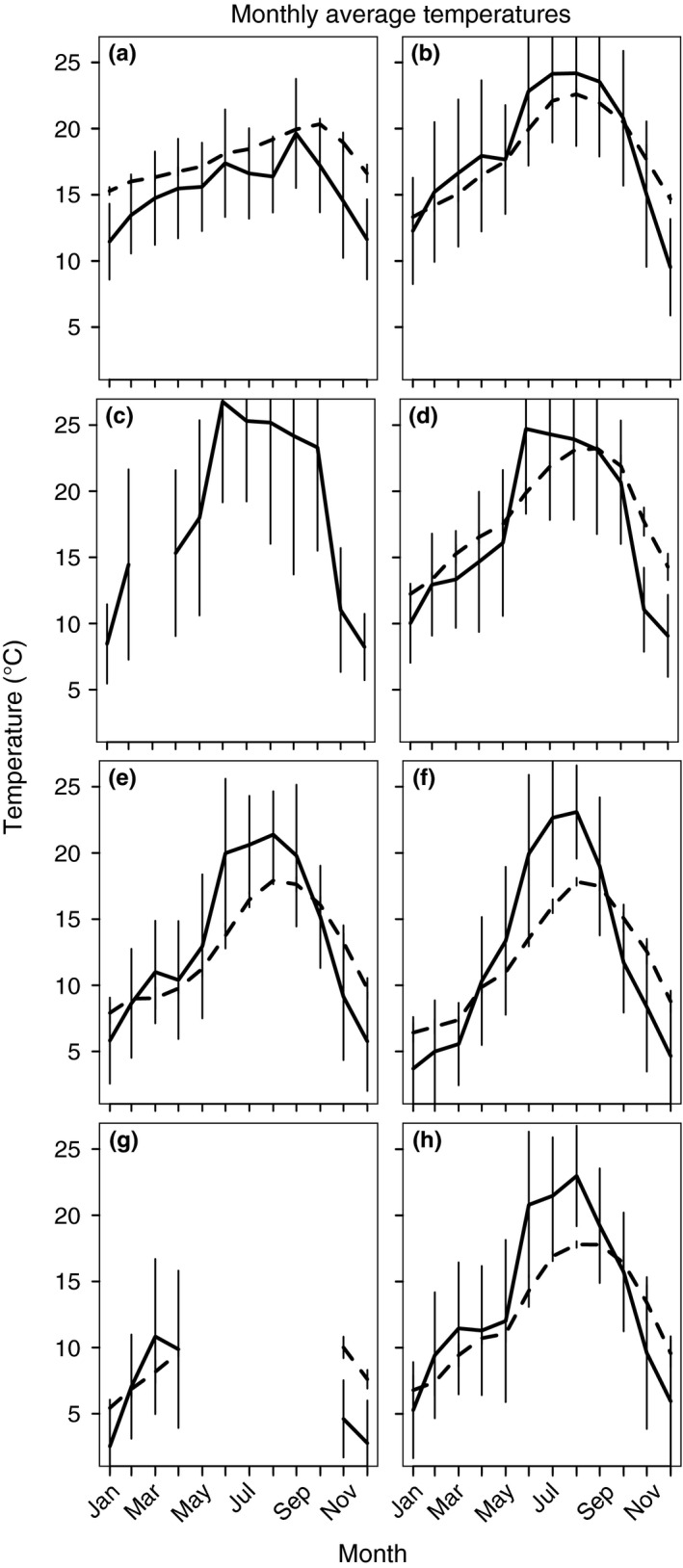
Monthly mean temperatures **(**±*SE*) both above ground (solid lines) and 60 cm below ground (dashed lines). (a) Berkeley, (b) Whittier, (c) Stebbins, (d) Quail Ridge, (e) Castle Rock, (f) Mt. Diablo, (g) Yosemite, and (h) Palomar Mtn

Approximately 250 worker ants were collected from each test population across elevations ranging from 71 to 1,442 m above sea level (Figure 1, Table 1; Appendix S1). Average dry weight of the individuals ranged from 0.78 to 1.28 mg (Table 1). The mean underground temperatures over 12 months (3,876–22,509 hourly readings) ranged from 11.9 to 18.1°C (Figure 2; Appendix S2; Berkeley; 17.8°C, Whittier; 18.0°C, Quail Ridge; 18.1°C, Castle Rock; 12.6°C, Mt. Diablo; 11.9°C, Palomar Mtn.; 12.6°C). The mean aboveground temperatures over 12 months (7,749–33,984 hourly readings) ranged from 12.3 to 18.3°C (Figure 2; Appendix S3; Berkeley; 15.4°C, Whittier; 18.3°C, Quail Ridge; 17.0°C, Castle Rock; 13.4°C, Mt. Diablo; 12.3°C, Palomar Mtn.; 13.8°C).

### 3.1 Chill‐coma recovery

**FIGURE 3:**
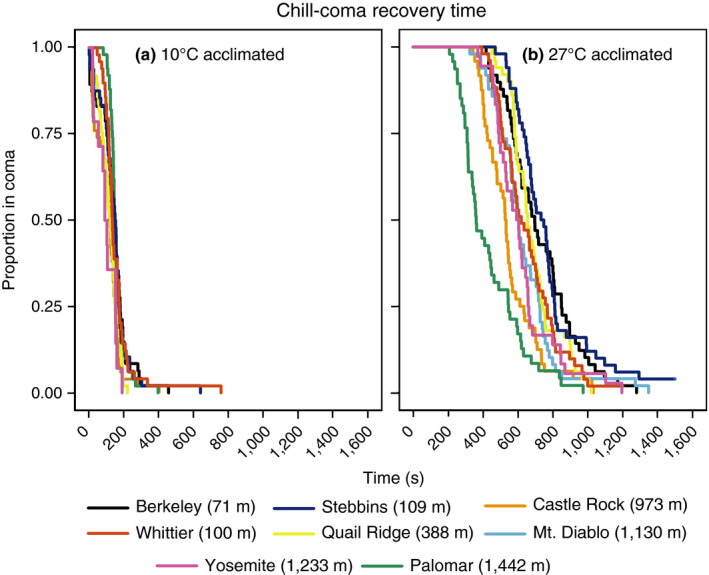
Chill‐coma recovery time for populations of *Prenolepis imparis*. (a) Chill‐coma recovery times after acclimation to 10°C and (b) chill‐coma recovery times after acclimation to 27°C. Individuals were pooled and represented by single lines colored according to population of origin

After three hours of being surrounded by ice, all ants were in chill coma. The most parsimonious model for CCRT was the Weibull error distribution. Chill‐coma recovery was influenced by acclimation temperature and population of origin (biological replicate as random variable; χ^2^_4_ = 2.66, *p* = .62). The strongest effect was from acclimation temperature, indicating a strong environmental effect: Individuals acclimated to 10°C recovered faster than those acclimated to 27°C (χ^2^_1_ = 124.99; *p* < .0001). Among‐population variance was higher than that of within‐population, indicating a genetic effect as well (population; χ^2^_7_ = 43.44; *p* < .0001; population:acclimation; χ^2^_7_ = 18.02; *p* < .05). Ants from all eight populations recovered faster when acclimated to the low temperature (Figure 3; Table 2). In addition, several populations (7/28) displayed different CCRT after the low versus the high‐temperature acclimation (6/28) (Table 2).

**Table 2:** Post hoc contrasts within and between populations of *Prenolepis imparis* chill‐coma recovery time after acclimation to 10°C (low acclimation) and 27°C (high acclimation)

Comparison	Fixed factor	β ± *SE*	*z*	*p*‐Value
Whittier–Quail Ridge	Low acclimation	0.44 ± 0.09	4.87	<.0001
Whittier–Castle Rock	Low acclimation	0.38 ± 0.09	4.24	<.001
Whittier–Mt Diablo	Low acclimation	0.42 ± 0.09	4.70	<.0001
Whittier–Yosemite	Low acclimation	0.49 ± 0.13	3.62	.0246
Stebbins–Quail Ridge	Low acclimation	0.38 ± 0.09	4.24	<.001
Stebbins–Castle Rock	Low acclimation	0.32 ± 0.09	3.59	.029
Stebbins–Mt Diablo	Low acclimation	0.36 ± 0.09	4.08	<.01
Berkeley–Palomar	High acclimation	0.46 ± 0.09	5.25	<.0001
Whittier–Palomar	High acclimation	0.37 ± 0.09	4.16	<.001
Stebbins–Castle Rock	High acclimation	0.35 ± 0.09	3.88	<.001
Stebbins–Palomar	High acclimation	0.54 ± 0.09	6.10	<.0001
Quail Ridge–Palomar	High acclimation	0.39 ± 0.09	4.46	<.001
Mt Diablo–Palomar	High acclimation	0.32 ± 0.09	3.66	.023
Berkeley	Low/high acclimation	−1.52 ± 0.09	−17.03	<.0001
Whittier	Low/high acclimation	−1.23 ± 0.09	−13.86	<.0001
Stebbins	Low/high acclimation	−1.47 ± 0.09	−16.22	<.0001
Quail Ridge	Low/high acclimation	−1.70 ± 0.09	−19.55	<.0001
Castle Rock	Low/high acclimation	−1.45 ± 0.09	−16.43	<.0001
Mt Diablo	Low/high acclimation	−1.61 ± 0.09	−18.47	<.0001
Yosemite	Low/high acclimation	−1.68 ± 0.14	−12.29	<.0001
Palomar	Low/high acclimation	−1.04 ± 0.09	−11.89	<.0001

Notes

Population and acclimation temperature were included as fixed factors, the replicates were included as random factors and differences are given as *β* ± *SE* standard error. Only pairs that were significantly different are shown.

**FIGURE 4:**
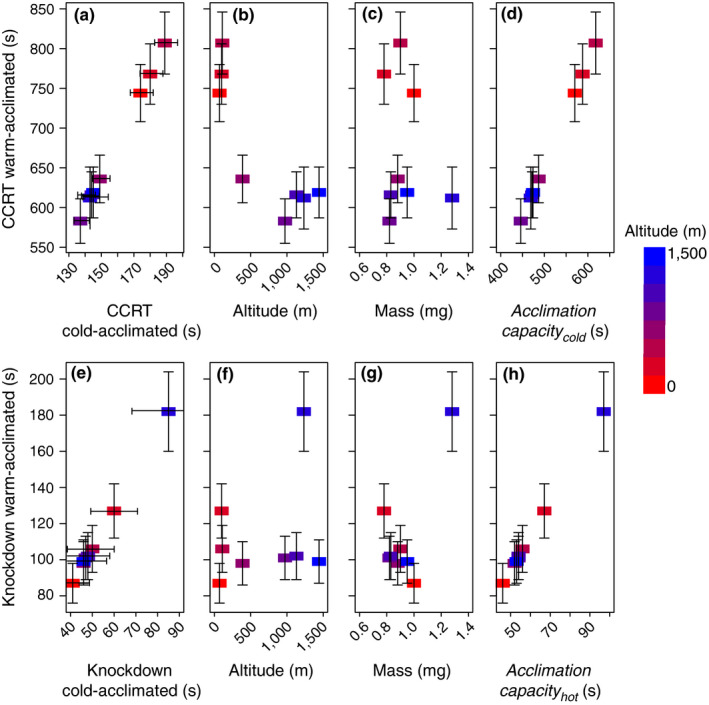
Modeled thermal tolerance after a high‐temperature acclimation (s) in relation to modeled low‐temperature acclimation (s), altitude (m), mass (mg), and acclimation capacity (s). (a) Chill‐coma recovery time (CCRT) after 27°C acclimation with respect to CCRT after 10°C acclimation. (b) CCRT with respect to altitude. (c) CCRT with respect to mass. (d) CCRT with respect to *acclimation capacity_cold_*. (e) Knockdown after 27°C acclimation with respect to knockdown after 10°C acclimation. (f) Knockdown time with respect to altitude. (g) Knockdown time with respect to mass. (h) Knockdown time with respect to *acclimation capacity_warm_*

Our estimator of thermal plasticity, *acclimation capacity_cold_*, ranged from 446 to 618s. Chill‐coma recovery after 27°C and CCRT after 10°C were highly correlated (Figure 4a; *R* ^2^ = 1.0, *F*_1,6_ = 36,090, *p* < .0001); therefore, we used only CCRT after 27°C for the following comparisons. Chill‐coma recovery was correlated with elevation (Figure 4b; *R* ^2^ = 0.72, *F*_1,6_ = 15.31, *p* = .008) but not with body size (Figure 4c; *R* ^2^ = 0.037, *F*_1,6_ = 0.23, *p* = .65), there was a strong relationship was between CCRT (i.e., thermal tolerance) and *acclimation *capacity_cold_ indicating that high tolerance was significantly correlated with high plasticity (Figure 4d; *R* ^2^ = 1.0, *F*_1,6_ = 669,000, *p* < .00001). Ants from the high‐elevation populations were characterized by faster CCRT times, indicating greater cold tolerance, than ants from low‐elevation sites. This was true for both hot‐ and cold‐acclimated ants.

### 3.2 Heat knockdown trials

**FIGURE 5:**
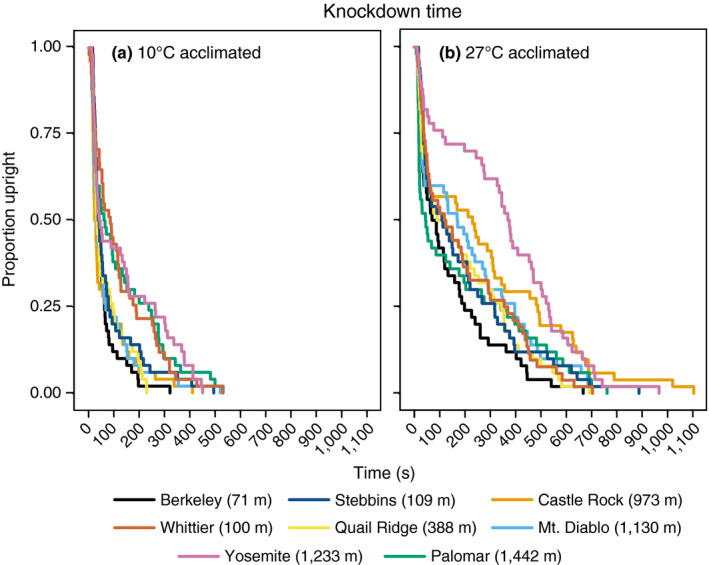
Knockdown for populations of *Prenolepis imparis*. (a) Knockdown times after acclimation to 10°C and (b) knockdown times after acclimation to 27°C. Individual knockdown times were pooled and colored according to population of origin

All ants succumbed to heat stress. The most parsimonious model for the knockdown trials was the lognormal error distribution. Knockdown time was influenced by acclimation temperature and population of origin (biological replicate as random variable; χ^2^_4_ = 0.019, *p* = .99). The strongest effect from was due to acclimation temperature, indicating a strong environmental effect: Individuals acclimated to 10°C experienced knockdown faster than those acclimated to 27°C (χ^2^_1_ = 81.11 *p* < .0001). Among‐population variance was higher than within‐population, indicating a genetic effect as well (population; χ^2^_7_ = 26.833; *p* < .001; population:acclimation; χ^2^_7_ = 18.94; *p* < .01). Six populations showed significant improvements in knockdown time after acclimation to 27°C (Figure 5; Table 3), but few population differences (3/28 after 10°C acclimation and 1/28 after 27°C) indicating much less among‐population variability (Table 3).

**Table 3:** Post hoc contrasts within and between populations of *Prenolepis imparis* knockdown time after acclimation to 10°C (low acclimation) and 27°C (high acclimation)

Comparison	Fixed factor	*β* ± *SE*	*z*	*p*‐Value
Berkeley–Whittier	Low acclimation	−0.72 ± 0.21	−3.46	.04
Berkeley–Yosemite	Low acclimation	−0.83 ± 0.21	−3.94	<.001
Berkeley–Palomar	Low acclimation	−0.79 ± 0.21	−3.76	.02
Berkeley–Yosemite	High acclimation	−0.93 ± 0.21	−4.46	<.0001
Berkeley	Low/high acclimation	−0.89 ± 0.21	−4.23	<.001
Stebbins	Low/high acclimation	−0.88 ± 0.20	−4.18	<.001
Quail Ridge	Low/high acclimation	−1.01 ± 0.21	−4.84	<.0001
Castle Rock	Low/high acclimation	−1.42 ± 0.21	−6.79	<.0001
Mt Diablo	Low/high acclimation	−1.10 ± 0.21	−5.26	<.0001
Yosemite	Low/high acclimation	−0.99 ± 0.21	−4.73	<.0001

Notes

Population and acclimation temperature were included as fixed factors, the replicates were included as random factors, and differences are given as *β* ± *SE* standard error. Only pairs that were significantly different are shown.

The mean acclimation capacity_hot_ for knockdown times ranged from 46 to 97s. Knockdown time after acclimation to 27°C was significantly correlated with knockdown time after 10°C (Figure 4e; *R* ^2^ = 1.00, *F*_1,6_ = 11,220, *p* < .0001); therefore, all comparisons were made with the knockdown time after acclimation to 27°C. The population knockdown means did not correlate with elevation (Figure 4e; *R* ^2^ = 0.08, *F*_1,6_ = 0.55, *p* = .49) nor body size (Figure 4f; *R* ^2^ = 0.49, *F*_1,6_ = 5.68, *p* = .054); yet, as with CCRT, the strongest relationship appeared to be between the mean *acclimation capacity_hot_* and knockdown after warm acclimation (Figure 4h; *R* ^2^ = 0.99, *F*_1,6_ = 3,192, *p* < .0001). We observe a similar pattern as we saw with CCRT, higher tolerance to heat, as shown by slower knockdown time, is significantly correlated with higher acclimation capacity or plasticity. There was no difference in heat tolerance (knockdown time) between ants from low‐elevation and high‐elevation sites, for both acclimated treatments; instead, greater tolerance correlated with greater plasticity.

## 4 DISCUSSION

The vulnerability of a taxon to escalating temperatures depends on its ability to buffer those changes, largely through phenotypic plasticity (Chevin, Lande, & Mace, 2010; Chown et al., 2010; Gunderson & Stillman, 2015; Huey et al., 2012; Somero, 2010; Williams, Shoo, Isaac, Hoffmann, & Langham, 2008). Here, we studied whether there are differences in thermal tolerance or plasticity among natural populations of the winter ant, *P. imparis*, a thermally sensitive ant. We found differences among populations in both thermal tolerance and plasticity at their upper and lower thermal limits.

Almost all populations were able to physiologically adjust to conditions after warm or cold acclimation. While there was greater cold tolerance in high altitude populations (i.e., more rapid CCRT at higher altitude), heat tolerance (knockdown time) was not correlated with elevation (Figure 4b, f). We also found increased tolerance in populations that also had increased plasticity for both cold and heat (Figure 4d, h).

### 4.1 Chill‐coma recovery

We observed that most populations had similar CCRT survivorship curves when they were acclimated to cold beforehand (10°C). There were a few population combinations (7/28) which we were able to detect changes after the low‐temperature acclimation: The differences were primarily observed in Whittier, Stebbins, or Palomar population. This adaptive phenotype allows cold‐exposed ants to endure even colder weather. Quick recovery is expected in cold‐tolerant species. For such species, David, Gibert, Moreteau, Gilchrist, and Huey (2003) reported an instant recovery to 0°C and recommended exposure to lower temperatures. After warm acclimation (27°C), individuals took longer to recover from the cold stress, presumably due to its magnification after higher temperature endurance. Previous studies have demonstrated similar results regarding linear associations between acclimation temperature and cold tolerance (Noh, Everman, Berger, & Morgan, 2017; Overgaard, Kristensen, Mitchell, & Hoffmann, 2011; Schou, Mouridsen, Sørensen, & Loeschcke, 2017).

Interestingly, CCRT after warm acclimation allowed for separation of different population combinations (6/28) indicating that different physiological mechanisms are employed to recover after a larger variation in temperature. The different patterns of response to CCRT can be explained by two different recovery methods driving the same trait. Insects will enter a chill coma as a result of a loss of ion balance within the central nervous system (CNS), which leads to neuromuscular paralysis (Andersen, Jensen, Meldrum Robertson, & Overgaard, 2018; Andersen & Overgaard, 2019). CCRT after prolonged or severe cold stress is thought to be related to the ability of insects to restore the ion balance in the hemolymph, and for insects that are acclimated to or tolerant of the cold, there is not a significant depolarization of muscles, resulting in a quick recovery of the depolarization via the CNS. For insects that are acclimated to warm temperatures or sensitive to the cold, there is more depolarization and loss of muscle function; consequently, the recovery is delayed from slower muscle function restoration (Andersen & Overgaard, 2019).

Chill‐coma recovery is correlated with collection altitude. Altitudinal clines for thermal tolerance have previously been described (Bishop, Robertson, Rensburg, & Parr, 2017; Castañeda et al., 2005; David et al., 2003; Hoffmann, Anderson, & Hallas, 2002), and thermal minima have been shown to vary more across environmental gradients than thermal maxima (Addo‐Bediako, Chown, & Gaston, 2000; Araújo et al., 2013). Temperature shifts associated with increasing latitude are the prevailing factors assumed to be the major selective force behind these clines. Our data show an inverse relationship between acclimation capacity_cold_ and elevation, suggesting thermal adaptation and underlying genetic variation (Bahrndorff et al., 2009; Calosi, Bilton, Spicer, Votier, & Atfield, 2010; Hoffmann et al., 2003; Schilthuizen & Kellermann, 2014; Sørensen, Dahlgaard, & Loeschcke, 2001). Previous studies have shown lower thermal limits reveal important ecological variation (Andersen et al., 2015; Bishop et al., 2017), and at least for one species of ant, maximum temperature of the warmest month and precipitation during the warmest quarter predicted the range boundary (Nguyen et al., 2019). Although we do not have enough statistical power to test for correlations between the acclimation capacity_cold_ and environmental data, our environmental data suggest a lower mean temperature at higher elevation.

We report here differences in CCRT as indicators of tolerance. This trait is complex (Sinclair et al., 2015), and future studies might revisit plasticity in these CCRT values with other measures of thermal tolerance such as chill‐coma temperature (CT_min_), lethal temperature (LTe_50_), or lethal time at low temperature (Lti_50_; Andersen et al., 2015). Though these traits have been shown to vary among insects, these additional measurements could be used to verify the conclusions presented here.

### 4.2 Knockdown trials

We found evidence for some intraspecific variation in tolerance at the upper thermal limit, similar to previous studies (Hoffmann et al., 2002; Sarup, Sørensen, Dimitrov, Barker, & Loeschcke, 2006). This pattern was different by the acclimation temperature and did not seem to be related to either altitude or body size. Although some studies have noted that upper thermal limits varied with altitude (Hoffmann et al., 2003), other studies have found no altitudinal variation in this trait (Arthur, Weeks, & Sgrò, 2008; Sarup et al., 2009; Slatyer, Nash, & Hoffmann, 2016). Previous estimates of heat resistance in arthropods suggest that upper thermal limits are evolutionarily constrained (Araújo et al., 2013; Deutsch et al., 2008; García‐Robledo, Kuprewicz, Staines, Erwin, & Kress, 2016; Gilchrist & Huey, 1999; Hoffmann et al., 2013) with acclimation conferring small plastic changes (Gunderson & Stillman, 2015; Kingsolver & Huey, 1998; Overgaard et al., 2011), and other times a heat‐acclimation response has been unpredictable (Schou et al., 2017). Bishop et al. (2017) found more pronounced differences in tolerances in lower thermal limits when compared to upper thermal limits similar to this study and suggest this could be due to more pronounced differences in lower temperatures compared to differences found in higher temperatures. Detection of an adaptive basis of temperature tolerance could also depend on methods used, and recent studies recommend using thermal death time (TDT) curves as a more complete and reliable measurement of heat tolerance (Castañeda, Rezende, & Santos, 2015; Jørgensen, Malte, & Overgaard, 2019). The difference in detecting more population differences in chill‐coma recovery versus fewer differences during knockdown time might represent a constraint that exists between the two types of plasticity. A cold‐specialized insect may need to physiologically extend their tolerance to the cold more often than it is necessary to adjust to the heat in addition to an evolutionary constraint in upper thermal limits.

We did not find evidence for an evolutionary trade‐off between basal thermal tolerance and plasticity in the context of knockdown time. Our data suggest a positive relationship; populations that had higher thermal tolerance also had greater acclimation capacity. This relationship has been observed in other species such as *Deronectes* beetles (Calosi et al., 2008), which could be driven by differences in gill density (Verberk, Calosi, Spicer, Kehl, & Bilton, 2018).

Acclimation to the heat increased the time it took to knockdown in six of the eight populations (Table 3). A pattern of low acclimation capacity to the heat has also been found in other studies, which further supports the idea that acclimation and adaptation to high temperatures are more challenging than acclimation and adaptation to low temperatures (Addo‐Bediako et al., 2000; García‐Robledo et al., 2016; Gunderson & Stillman, 2015; van Heerwaarden et al., 2016; Kingsolver & Huey, 1998; Overgaard et al., 2011; Stillman, 2003). This short‐term plasticity has been shown to be an important adaptive mechanisms to cope with extreme temperatures (Chidawanyika & Terblanche, 2011; Kellett et al., 2005; Mitchell, Sgrò, & Hoffmann, 2011), though the potential of phenotypic plasticity to completely buffer individuals from heat stress is limited.

## 5 CONCLUSIONS

It is still unclear how thermal tolerance of the *P. imparis* workers correlates with overall fitness of the colony or the queen, and how this relationship between tolerance and activity plays out in natural settings. Given their low tolerance of high temperatures, these ants will need to behaviorally thermoregulate in order to survive climate warming (Sunday et al., 2014) by exploiting their small size to take advantage of thermal heterogeneity in the environment. Hemmings and Andrew (2017) found that ants in environments that exceed their thermal tolerance could maintain lower body temperatures than the surrounding temperatures, suggesting that they are using unknown behavioral or physiological methods to achieve homeostasis.

The localized patterns of plasticity in populations of *P. imparis* could be due to microclimate differences or genetic differences or maternal effects. Our experimental design does not allow us to distinguish the processes behind the patterns. The importance of these factors could be resolved using a common garden experiment, in which multiple generations are reared under controlled lab conditions (Hoffmann & Sgrò, 2017; Kawecki & Ebert, 2004). By doing this, we will better be able to understand the role of environmental effects of genetic variation. Tolerance to stressful temperatures in ectotherms, especially resistance to cold extremes, is strongly affected by environmental variation (Ørsted, Hoffmann, Rohde, Sørensen, & Kristensen, 2018; Schou et al., 2017).

Here, we have shown that individuals from different populations of the winter ant show varying levels of thermal tolerance at both upper and lower thermal temperatures, and most populations display less tolerance and reduced plasticity to the high temperatures. This is particularly concerning in the context of climate change. Historically, winter ants might have been able to avoid extensive exposure to the heat by remaining below ground during the times of hottest temperature. In a warmer future climate, however, they may have to spend even more time belowground, limiting their ability to forage, and thus, to persist. Our study may provide insight into evolution and creation of biodiversity by revealing physiological variance both within and between populations. As evidenced above, it is paramount to consider variation within species when attempting to understand how species distributions are affected by thermal extremes.

## CONFLICT OF INTEREST

The authors declare no conflict of interest.

## AUTHOR CONTRIBUTION

**Maria Adelena Tonione:** Conceptualization (lead); Formal analysis (lead); Investigation (lead); Visualization (lead); Writing‐original draft (lead). **So Mi Cho:** Conceptualization (supporting); Data curation (supporting); Investigation (supporting); Writing‐review & editing (supporting). **Gary Matthew Richmond:** Data curation (lead); Investigation (supporting); Writing‐review & editing (supporting). **Christian George Irian:** Data curation (supporting); Investigation (supporting); Writing‐review & editing (supporting). **Neil Durie Tsutsui:** Conceptualization (supporting); Investigation (supporting); Methodology (supporting); Writing‐review & editing (supporting). 

## Supporting information

Tables S1–S3

## Data Availability

The doi for our data is https://doi.org/10.6078/D1KM48.
