# Supplementary material for: Intraspecific variation in thermal acclimation and tolerance between populations of the winter ant, Prenolepis imparis
Source: Ecol Evol. 2020 Apr 8;10(11):4749–61. doi: 10.1002/ece3.6229 (PMC7297759; doi:10.1002/ece3.6229)
Supplement: Supplementary file 1 — Tables S1–S3 [file ECE3-10-4749-s001.docx]

**Intraspecific variation in thermal acclimation and tolerance between populations of the winter ant, *Prenolepis imparis***

Maria Adelena Tonione^a,^*, So Mi Cho^a,1^, Gary Richmond^a^, Christian Irian^a^, and Neil Durie Tsutsui^a^

**Supplemental Table S1** Locality name, altitude (m), GPS coordinates (longitude and latitude), and mean mass are given for *P. imparis* populations sampled. For each trial total number sampled as well as number of biological replicates and the range of individuals per replicate in parentheses for each acclimation temperature is also given.

|  |  |  | **CCRT** | | | | **knockdown** | | | |
| --- | --- | --- | --- | --- | --- | --- | --- | --- | --- | --- |
| **Locality** | **Longitude** | **Latitude** | **n**  **10ºC** | **br** | **n**  **27ºC** | **br** | **n**  **10ºC** | **br** | **n**  **27ºC** | **br** |
| Berkeley | -122.26317 | 37.87281 | 47 | 5  (8-10) | 49 | 5  (9-10) | 50 | 5 (10) | 50 | 5 (10) |
| Whittier | -118.05395 | 34.00381 | 49 | 5  (9-11) | 51 | 5  (10-11) | 51 | 5  (10-11) | 52 | 5  (9-13) |
| Stebbins Cold Canyon Reserve | -122.09678 | 38.50867 | 48 | 5  (9-10) | 50 | 5 (10) | 50 | 5 (10) | 50 | 5 (10) |
| Quail Ridge Reserve | -122.14895 | 38.48307 | 49 | 5  (9-11) | 50 | 5 (10) | 50 | 5 (10) | 50 | 5 (10) |
| Castle Rock State Park | -122.09495 | 37.22829 | 50 | 5 (10) | 48 | 5  (8-10) | 50 | 5 (10) | 51 | 5  (10-11) |
| Mt. Diablo State Park | -121.916667 | 37.219167 | 49 | 5  (9-10) | 49 | 5  (8-11) | 50 | 5 (10) | 50 | 5 (10) |
| Yosemite National Park | -119.58584 | 37.74763 | 14 | 2  (4-10) | 36 | 4  (7-10) | 50 | 5 (10) | 50 | 5 (10) |
| Palomar Mountain State Park | -116.92146 | 33.34078 | 50 | 5 (10) | 47 | 5  (8-10) | 50 | 5 (10) | 50 | 5 (10) |

n = total number of individuals tested

br = total number of biological replicates, with the range of the number of individuals in parentheses

**Supplemental Table S2** Mean underground temperatures for sampled localities of *P. imparis*. Temperature reported in °C (± SD).

| **Locality** | **Temp. readings** | **Jan** | **Feb** | **Mar** | **Apr** | **May** | **Jun** | **Jul** | **Aug** | **Sep** | **Oct** | **Nov** | **Dec** |
| --- | --- | --- | --- | --- | --- | --- | --- | --- | --- | --- | --- | --- | --- |
| Berkeley | 18,275 | 11.5 (2.9) | 13.5 (2.9) | 14.8 (3.5) | 15.5 (3.8) | 15.6 (3.3) | 17.4 (4.1) | 16.6 (3.4) | 16.4 (2.7) | 19.7 (4.1) | 17.2 (3.5) | 14.6 (4.3) | 11.7 (3.0) |
| Whittier | 33,984 | 12.3 (4.0) | 15.2 (5.3) | 16.7 (5.6) | 18.0 (5.7) | 17.7 (4.1) | 22.8 (5.6) | 24.2 (5.2) | 24.2 (5.5) | 23.6 (5.7) | 20.8 (5.1) | 15.1 (5.5) | 9.5 (3.6) |
| Stebbins Cold Canyon Reserve | 24,123 | 8.5 (3.0) | 14.5 (7.2) |  | 15.3 (6.3) | 18.0 (5.7) | 26.8 (7.6) | 25.3 (6.1) | 25.2 (9.2) | 24.2 (10.5) | 23.3 (7.8) | 11.0 (4.7) | 8.2 (2.5) |
| Quail Ridge Reserve | 15,114 | 10 (3.0) | 12.9 (3.9) | 13.3 (3.7) | 14.7 (5.3) | 16.1 (5.5) | 24.7 6.4) | 24.3 (6.5) | 23.9 (6.1) | 23.2 (6.4) | 20.7 (4.7) | 11 (3.2) | 9.1 (3.1) |
| Mt. Diablo Sate Park | 19,869 | 3.7 (3.9) | 5 (3.9) | 5.6 (3.1) | 10.3 (4.8) | 13.4 (5.6) | 19.9 (6.0) | 22.7 (5.2) | 23.1 (3.5) | 19 (5.2) | 11.7 (3.8) | 8.4 (4.9) | 4.6 (4.1) |
| Yosemite National Park | 16,384 | 2.5 (3.5) | 7.1 (3.9) | 10.8 (5.9) | 9.9 (5.9) |  |  |  |  |  |  | 4.6 (2.9) | 2.8 (3.2) |
| Palomar Mountain State Park | 19,853 | 5.3 (3.6) | 9.4 (4.8) | 11.5 (5.0) | 11.3 (4.9) | 12 (6.1) | 20.8 (5.5) | 21.5 (4.4) | 23.0 (3.8) | 19.2 (4.3) | 15.7 (4.5) | 9.6 (5.7) | 5.9 (4.9) |
| Castle Rock | 7,749 | 5.8 (3.3) | 8.6 (4.1) | 11 (3.9) | 10.4 (4.5) | 12.9 (5.4) | 20.0 (5.6) | 20.6 (3.7) | 21.4 (3.3) | 19.8 (5.4) | 15.2 (3.9) | 9.1 (4.8) | 5.8 (3.7) |

**Supplemental Table S3** Mean above ground temperatures for sampled localities of *P. imparis*. Temperature reported in °C (± SD).

| **Locality** | **Temp. readings** | **Jan** | **Feb** | **Mar** | **Apr** | **May** | **Jun** | **Jul** | **Aug** | **Sep** | **Oct** | **Nov** | **Dec** |
| --- | --- | --- | --- | --- | --- | --- | --- | --- | --- | --- | --- | --- | --- |
| Berkeley | 10,072 | 15.3 (0.3) | 16.0 (0.5) | 16.3 (0.2) | 16.8 (0.3) | 17.2 (0.0) | 18.1 (0.1) | 18.5 (0.3) | 19.2 (0.2) | 20 .0 (0.2) | 20.4 (0.2) | 19.0 (0.7) | 16.6 (0.7) |
| Whittier | 20,945 | 13.3 (0.4) | 14.2 (0.5) | 15.1 (0.6) | 16.5 (0.5) | 17.5 (0.4) | 20.0 (1.1) | 22.1 (0.5) | 22.6 (0.4) | 22.0 (0.4) | 20.5 (0.6) | 17.7 (1.1) | 14.7 (0.3) |
| Quail Ridge Reserve | 19,709 | 12.2 (0.6) | 13.3 (0.5) | 15.3 (0.7) | 16.5 (0.5) | 17.5 (0.5) | 19.9 (0.8) | 21.9 (0.4) | 23.1 (0.3) | 23.2 (0.3) | 21.9 (0.8) | 17.7 (1.1) | 14.3 (1.0) |
| Mt. Diablo State Park | 8,192 | 6.4 (0.7) | 6.9 (0.5) | 7.4 (0.2) | 9.9 0.5) | 11 (0.5) | 13.5 (0.6) | 16.0 (0.5) | 17.8 (0.3) | 17.5 (0.4) | 15.1 (1.0) | 12.6 (1.0) | 8.8 (0.8) |
| Yosemite National Park | 3,876 | 5.4 (0.2) | 6.9 (0.6) | 8.2 (0.9) | 9.6 (0.3) |  |  |  |  |  |  | 10.0 (0.8) | 7.6 (0.7) |
| Palomar Mountain State Park | 21,177 | 6.8 (1.1) | 7.4 (1.0) | 9.4 (0.8) | 10.7 (0.5) | 11.1 (0.6) | 14.3 (1.2) | 16.9 (0.4) | 17.8 (0.2) | 17.8 (0.3) | 16.4 (0.5) | 13.4 (1.3) | 9.6 (1.0) |
| Castle Rock State Park | 22,509 | 7.9 (0.8) | 9.0 (0.6) | 9.0 (0.8) | 9.8 (0.4) | 11.3 (0.6) | 13.7 (1.0) | 16.5 (0.6) | 17.9 (0.3) | 17.6 (0.6) | 16.1 (0.6) | 13.3 (1.3) | 9.7 (0.8) |
